# Differentiation of suprasellar meningiomas from non-functioning pituitary macroadenomas by ^18^F-FDG and ^13^N-Ammonia PET/CT

**DOI:** 10.1186/s12885-020-06852-y

**Published:** 2020-06-17

**Authors:** Lei Ding, Fangling Zhang, Qiao He, Zhoulei Li, Xinchong Shi, Ruocheng Li, Xiangsong Zhang

**Affiliations:** 1 Department of the Medical Imaging, The First Affiliated Hospital, Sun Yat-sen University, 58# Zhongshan Er Road, Guangzhou, Guangdong Province 510080 People’s Republic of China; 2 Department of Radiology, Hospital of Stomatology, Guanghua School of Stomatology, Sun Yat-sen University; Guangdong Provincial Key Laboratory of Stomatology, 56#, Cemetery west Road, Guangzhou, Guangdong Province 510055 People’s Republic of China

**Keywords:** Non-functioning pituitary macroadenoma, Suprasellar meningioma; ^18^F-FDG; ^13^N-ammonia; PET-CT

## Abstract

**Background:**

Differentiation of suprasellar meningiomas (SSMs) from non-functioning pituitary macroadenomas (NFPMAs) is useful for clinical management. We investigated the utility of ^13^N-ammonia combined with ^18^F-FDG positron emission tomography (PET)/computed tomography (CT) in distinguishing SSMs from NFPMAs retrospectively.

**Methods:**

Fourteen NFPMA patients and eleven SSM patients with histopathologic diagnosis were included in this study. Every patient underwent both ^18^F-FDG and ^13^N-ammonia PET/CT scans. The tumor to gray matter (T/G) ratios were calculated for the evaluation of tumor uptake.

**Results:**

The uptake of ^18^F-FDG was higher in NFPMAs than SSMs, whereas the uptake of ^13^N-ammonia was obviously lower in NFPMAs than SSMs. The differences of ^18^F-FDG and ^13^N-ammonia uptake between the two groups were significant respectively (0.92[0.46] vs 0.59[0.29], *P* < 0.05, ^18^F-FDG; 1.58 ± 0.56 vs 2.80 ± 1.45, *P* < 0.05, ^13^N-ammonia). Tumor classification demonstrated a high overall accuracy of 96.0% for differential diagnosis. When the two traces were combined, only 1 SSM was misclassified into the NFPMA group.

**Conclusion:**

SSMs and NFPMAs have different metabolic characteristics on ^18^F-FDG and ^13^N-ammonia PET images. The combination of these two tracers can effectively distinguish SSMs from NFPMAs.

## Background

SSMs have been classified into three subtypes according to the original site and central location of the tumors: tuberculum sellae, planum sphenoidale and diaphragm sellae meningiomas [1–3]. The wide operative field of the trans-cranial approach is more appropriate for these firm tumors [2, 4]. Pituitary adenomas are the most common sellar region tumors and the surgery are conducted usually with the trans-sphenoidal approach [5]. SSMs can mimic other non-hormone secreting sellar region masses both clinically and radiologically, in particular the NFPMAs. In neurosurgical practice, it is important to distinguish SSMs from NFPMAs, which can result in differences in surgery program. Magnetic resonance imaging (MRI) is the most common neuroimaging modality owing to its excellent soft-tissue contrast and spatial resolution at present. In comparison with moderate and heterogeneous enhancement of NFPMAs, previous studies stated marked and homogeneous enhancement and “dural tail” sign with contrast as unique characteristics of SSMs [6, 7]. Moreover, mostly suprasellar center, moderate sella turcica enlargement and separation from pituitary gland may also be diagnostic elements for SSMs [8]. Nevertheless, accurate preoperative diagnosis is still quite challenging and difficult by current morphological imaging modalities alone because of the overlap of imaging findings and the relatively rarity of SSMs. MRI should be complemented by PET in whether diagnosis and staging before treatment or postoperative therapeutic effectiveness monitoring [9] .^13^N-ammonia is suggested to be a contrast-enhanced radiotracer which is more sensitive and specific than Gadolinium-Diethylenetriaminepentaacetic acid (Gd-DTPA) [10]. We have reported the clinical usefulness of ^13^N-ammonia in many brain tumors through a series of studies [11–14]. In addition, ^13^N-ammonia is also a potential tracer targeting glutamine synthetase (GS) expression which is associated with ammonia-glutamine synthesis reaction [15]. In this study, we aimed to investigate the combined efficiency of ^18^F-FDG and ^13^N-ammonia PET/CT in distinguishing SSMs from NFPMAs.

## Methods

### Patient

SSMs which broke through the diaphragma sellae and grew into the pituitary fossa and more than 1 cm in diameter, NFPMAs which grew anteriorly and superiorly and more than 1 cm in diameter were included in our study. Eventually the data of 25 patients in our center were enrolled in the study between July 2009 to December 2018. 14 patients with NFPMA (7 female and 7 male; mean age, 47.81 ± 10.04 years; range, 37–68 years) and 11 patients with SSM (9 female and 2 male; mean age, 55.69 ± 14.37 years; range, 41–83 years). All patients were absent of any therapeutic interventions before imaging examination and underwent PET/CT scan with ^18^F-FDG and ^13^N-ammonia within 5 days. Histopathological diagnosis was obtained after PET/CT scan for all cases. This study was approved by the hospital ethics committee. Detailed study purpose and imaging procedure were explained to every patient, and the need for signed informed consent was waived.

### PET/CT imaging

PET/CT scan was performed with a Gemini GXL-16 scanner (Philips, Netherlands) in 3-dimensional acquisition mode. ^18^F-FDG and ^13^N-ammonia were produced in our center using standard techniques and commercially available systems for isotope generation (Ion Beam Applications, Cyclone-10, Belgium). PET images were acquired by a particular imaging protocol for the brain with a field of view of 180 mm, reconstructed by the line of response algorithm and attenuation-corrected using low-dose CT. All patients fasted for at least eight hours and urinated just before PET/CT scan. About Forty-five minutes after an intravenous injection of ^18^F-FDG (5.18 MBq/kg) and five minutes after an intravenous injection of ^13^N-ammonia (370–740 MBq), a 10-min PET/CT scan started. ^18^F-FDG and ^13^N-ammonia studies were performed at least 24 h apart.

### Imaging analysis

#### Visual analysis

For visual analysis, the degree of tracer uptake by the lesion was visually classified into 3 grades compared with the contralateral or surrounding normal brain parenchyma: high metabolism, moderate metabolism and low metabolism.

#### Semiquantitative analysis

The uptake of the tumor was evaluated using the maximum standard uptake value (SUV_max_). For each patient, a region of interest (ROI) with 10 mm in diameter was drawn in the area of highest activity within the tumor in trans-axial plane. Then another reference ROI was placed on the normal contralateral gray matter of prefrontal cortex. The SUV_max_ of all ROIs were used for the calculation of T/G ratios. MRI images were referred to avoid the area of necrosis or hemorrhage by coregistering PET and MR images with the software of MIPAV (Center for Information Technology, National Institutes of Health, Maryland), especially in cases without significant tracer concentration.

### Statistical analysis

Statistical analysis was processed with SPSS 20.0 software (http://www.ibm.com). Result was considered statistically significant when the *P* value was less than 0.05. In this study, Student *t* test was firstly applied to compare the T/G ratios between NFPMA group and SSM group for each tracer. Then the T/G ratios of both tracers were used as multiple variables for the discrimination analysis of the two groups, generating the canonical discriminant function. As a result, each patient was classified into one group successfully according to the function result and cross validation was done to assess the differential usefulness when the two traces were combined.

## Results

All of the SSMs in our study are grade I referring to the 2016 World Health Organization (WHO) classification, including 7 meningothelial meningiomas, 3 transitional meningiomas and 1fibrous meningioma histopathologically. The diameter of NFPMAs and SSMs ranged from 1.2 to 5.8 cm (mean ± SD, 3.08 ± 1.25 cm) and 2.4 to 7.6 cm (mean ± SD, 3.37 ± 1.58 cm) respectively.

For NFPMAs, there were 2(14.3%), 8(57.1%), 4(28.6%) of the 14 cases showed low, moderate and high metabolism respectively on ^18^F-FDG images and 3(21.4%), 8(57.1%), 3(21.4%) of the 14 cases showed low, moderate and high metabolism respectively on ^13^N-ammonia images. For SSMs, there were 7(63.6%), 4(36.4%) of the 11 cases showed low and moderate metabolism respectively on ^18^F-FDG images and 11(100%) of the 11 cases showed high metabolism on ^13^N-ammonia images.

**Fig. 1:**
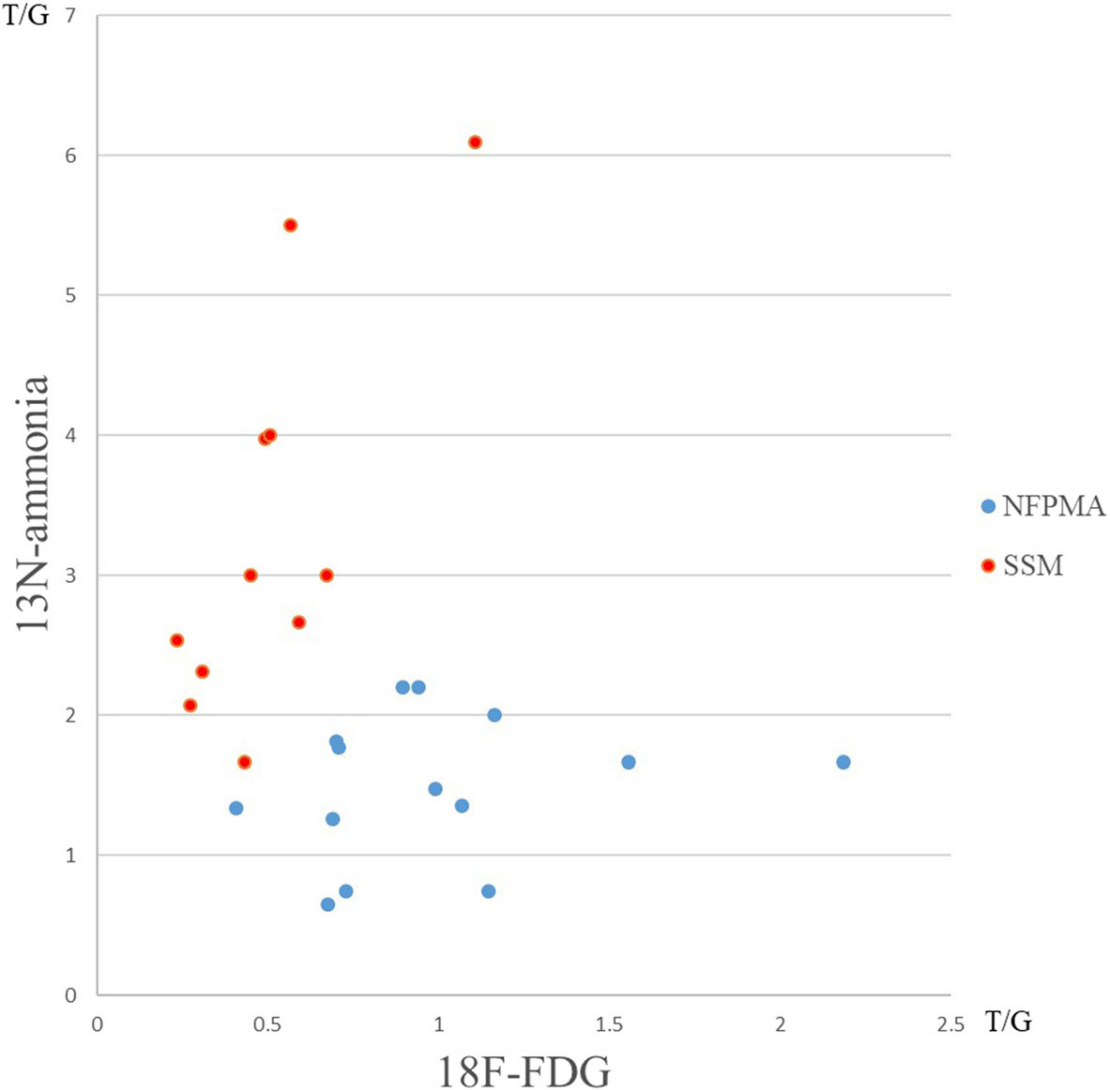
Distribution map of T/G ratios of ^18^F-FDG and ^13^N-ammonia. As shown in this figure, there was a small overlap between NFPMA (blue spot) and SSM (red spot)

**Fig. 2:**
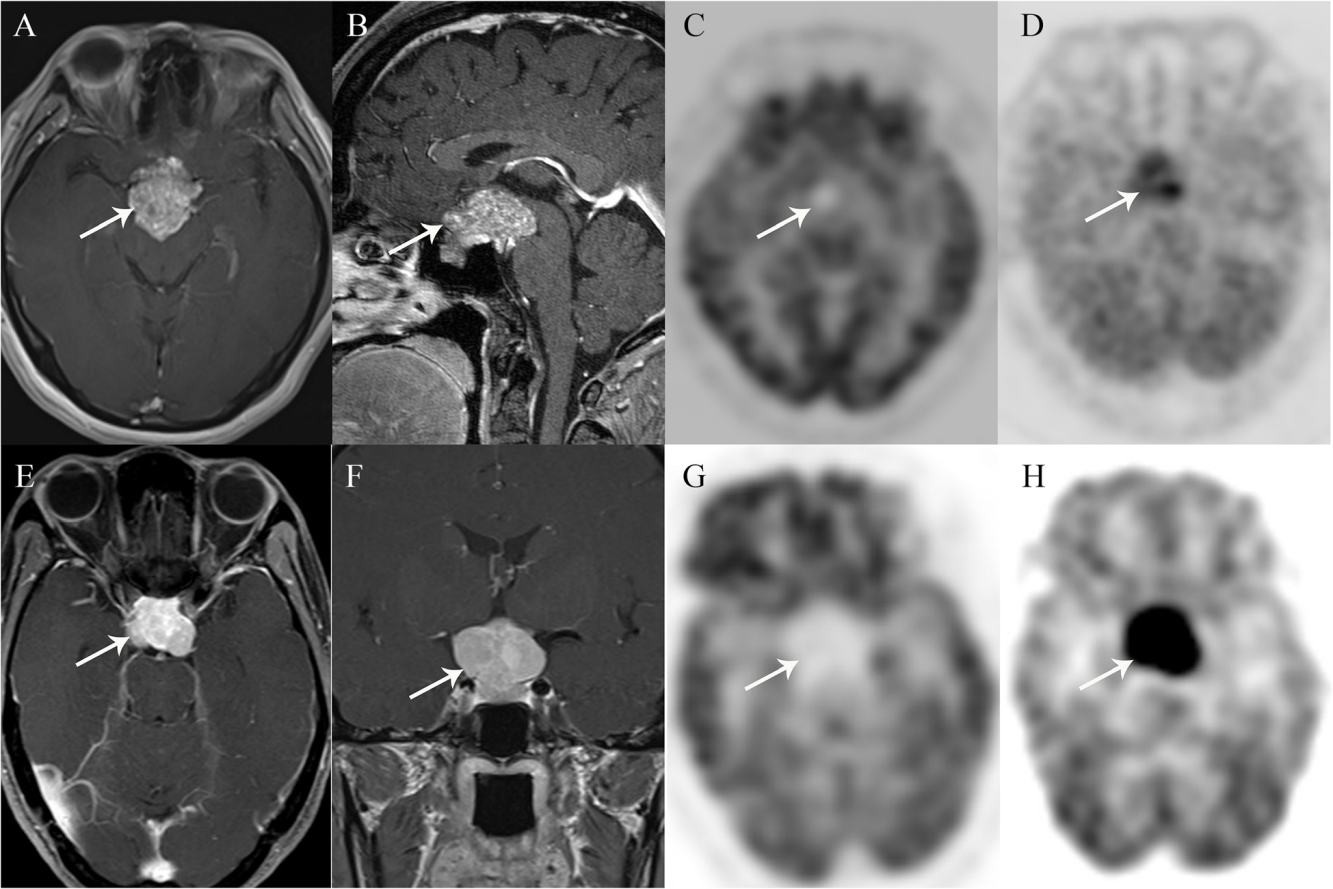
Imaging results in cases with NFPMA(A-D) and SSM(E-F). Trans-axial and sagittal contrast MRI images (**a**, **b**) showed the tumor (white arrows) had obvious enhancement. The pituitary gland was under the tumor intactly (white arrowheads) and the tumor was misdiagnosed as a pituitary macroadenoma on MRI images. Slight uptake of ^18^F-FDG (**c**) and high uptake of ^13^N-ammonia (**d**) on PET images. Trans-axial and coronal contrast MRI images (**e**, **f**) showed a marked enhancement (white arrows) mass mostly in the suprasellar region. The mass grew into the pituitary fossa and the pituitary gland was compressed. The lesion displayed obviously higher uptake of ^13^N-ammonia (**h**) compared with normal gray matter and low uptake of ^18^F-FDG (**g**)

**Fig. 3:**
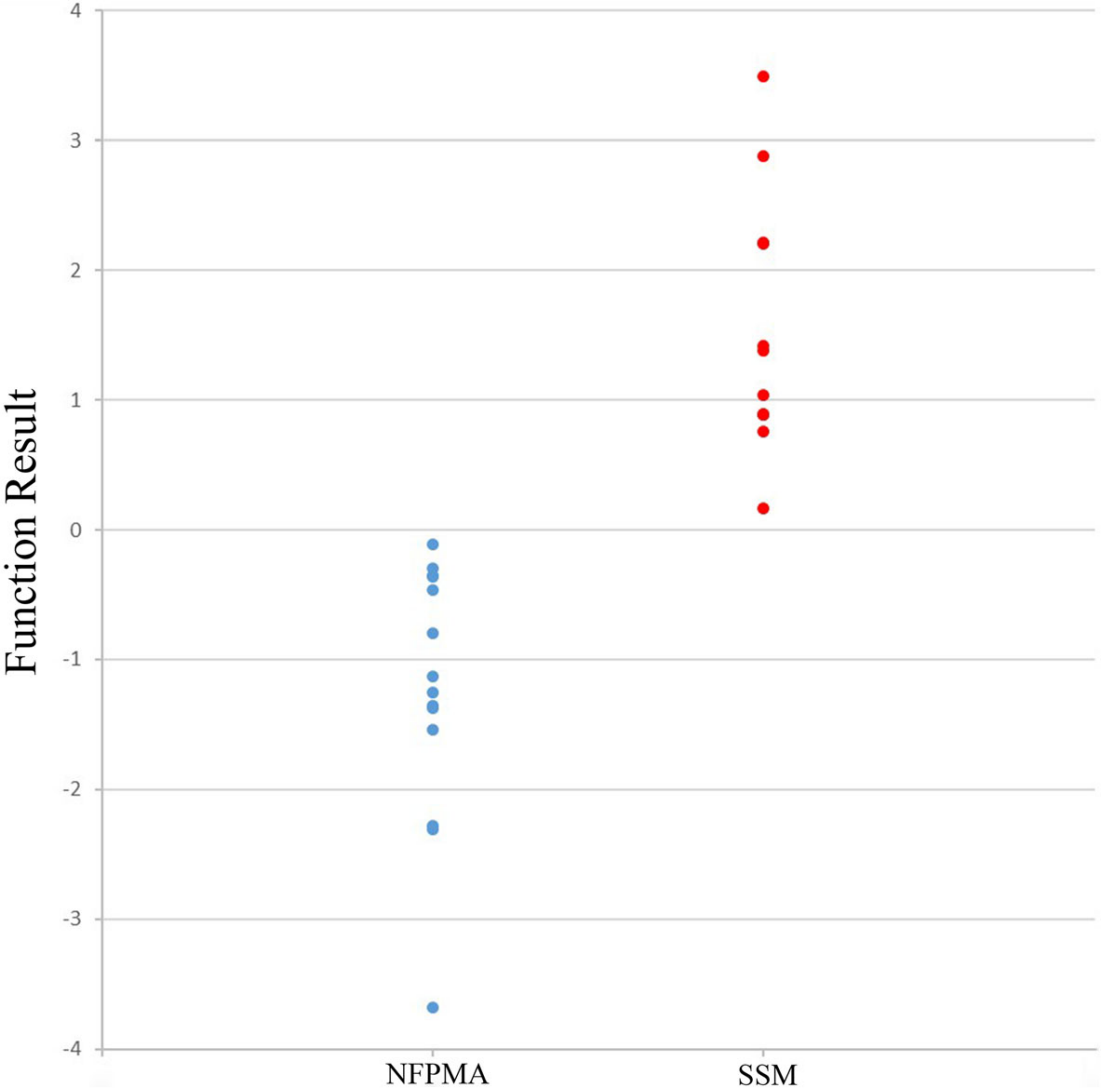
Discriminant function results of T/G ratios between the two groups. The function results of NFPMA were obviously lower than that of SSM (− 1.23 ± 0.96 vs 1.57 ± 1.01). The combination of the two tracers could distinguish these two clinical entities effectively

The uptake results were shown in Fig. 1. There were significant differences of ^18^F-FDG and ^13^N-ammonia uptake between the two clinical entities. The uptake of ^18^F-FDG was higher in NFPMAs than SSMs (0.92[0.46] vs 0.59[0.29], *P* < 0.05), whereas the concentration of ^13^N-ammonia was lower in NFPMAs than SSMs (1.58 ± 0.56 vs 2.80 ± 1.45, *P* < 0.05) (Fig. 2). Tumor classification by canonical discriminant analysis with T/G values of both tracers showed the optimal discriminant function was F (*x*, *y*) = − 2.191*x* + 0.946*y* – 0.473, where *x* represented T/G ratio of ^18^F-FDG and *y* represented T/G ratio of ^13^N-ammonia. Therefore, the function result of NFPMAs was − 1.23 ± 0.96, which was significantly lower than that of SSMs (1.57 ± 1.01, *P* < 0.001). The predicted accuracy for NFPMAs and SSMs was 100 and 90.9% respectively. Only 1 SSM was misdiagnosed as NFPMA and the overall diagnostic accuracy was 96.0% (Table 1, Fig. 3).

**Table 1:** Predicted accuracy of discriminant analysis for the 2 groups

		Predicted Group Membership			
		Group	NFPMA	SSM	Total
**Original**	n (%)	NFPMA	14 (100)		14 (100)
		SSM	1 (9.1)	10 (90.9)	11 (100)
**Cross-validated**	n (%)	NFPMA	14 (100)		14 (100)
		SSM	1 (9.1)	10 (90.9)	11 (100)

## Discussion

NFPMAs can cause hypopituitarism and hyperprolactinemia or even show no endocrinologic abnormalities, which is similar to SSMs [16]. Headaches, loss of visual acuity and visual field are some other common symptoms for these patients [7, 17]. Most intra-suprasellar adenomas are excised via the trans-sphenoidal route to our knowledge [18]. Unlike the soft pituitary adenomas, SSMs are highly vascular, firm and adhesive to adjacent neurovascular structures [19]. A transcranial approach is commonly considered for most SSMs, which can achieve a high rate of total resection with a low rate of postoperative cerebrospinal fluid leak, hemorrhage and nerve injury [4, 20, 21]. SSMs are sometimes amenable to resection by trans-sphenoidal route and some authors advocated that this approach can result in comparable outcomes with trans-cranial approach in carefully and critically selected patients [22, 23]. Generally speaking, the determination of an optimum surgical approach depends on multiple factors, such as tumor size, consistency, location, relationships with the adjacent structures and the presenting symptoms [24]. Therefore, surgical plan could be implemented more effectively and safely if an accurate pre-operative diagnosis is available.

MRI has played an irreplaceable role in the diagnosis of sellar and suprasellar tumors for a long time and most SSMs can be differentiated from NAPMAs based on it. Sometimes SSMs can break through the diaphragma sellae and grow into the pituitary fossa, making it similar to NFPMAs extending anteriorly and superiorly on conventional morphological imaging [7, 25]. Magnetic resonance brain functional imaging methods such as perfusion-weighted imaging (PWI) can be valuable for brain tumors by detecting the hemodynamic state. However, PWI is not always used in an efficient way. Firstly, PWI is highly user-dependent because the accurate recognition of blood vessels is challenging and the standardization is lacking in data processing methods presently. Besides, PWI is difficult to implemented in regions close to a brain–bone–air interface such as the skull base [26–28]. As one of the most important molecular imaging modalities, PET should be considered as complementary tools in the evaluation of brain tumors and PET even seems to show a more significant role than such MR advanced techniques [28, 29]. ^18^F-FDG is the most common PET/CT tracer and the molecular mechanism has been clarified previously [30]. However, the inherent limitation of high physiologic uptake in the normal brain tissue necessitates the search for other newer PET tracers. Although ^13^N-ammonia has a short half-life time, it is rapidly spreading in recent years because of its diagnostic power (perfusion-metabolism coupling tracer) and the easier interpretation for clinicians owing to the higher tumor to back-ground contrast compared to PWI and other PET tracers [31]. On the other hand, the short half-life time has the advantage of reducing the radiation. Actually, the synthesis time for ^13^N-ammonia is short and the process is convenient by the cyclotron. According to previous studies of our department, ^13^N-ammonia has potential diagnostic value in brain tumor [10, 11, 32, 33].

In our study, SSMs showed a higher uptake than NFPMAs on ^13^N-ammonia PET images. In contrast, NFPMAs showed a higher uptake than SSMs on ^18^F-FDG PET images. All SSMs are grade I, demonstrating lower glucose consumption than normal gray matter. The normal pituitary gland showed little uptake because of its small size and low metabolic rate on ^18^F-FDG PET images. On the other hand, pituitary adenomas were more metabolically active than normal pituitary gland and SSMs and the uptake was related to the size of the adenomas [34, 35]. ^18^F-FDG PET/CT was useful for detecting NFPMAs [36, 37]. For ^13^N-ammonia, we found it had great value in the diagnosis of SSMs. ^13^N-ammonia is lipid soluble and has small molecular weight (16 Da) compared to Gd-DTPA (approximately 470 Da) [38]. The blood ammonia mainly exists in two forms at physiological blood pH, that is unionized ammonia (NH_3_, about 3%) and ionized species (NH^+^_4_, about 97%). Compared with ionized form. The unionized form can pass the blood brain barrier (BBB) freely and be rapidly supplemented from the ionized form since the two forms are in equilibrium through the reaction NH^+^_4_ ↔ NH_3_ + H^+^ [39–41]. Actually, the initial extraction of ^13^N-ammonia depends on the cerebral blood flow (CBF) and capillary permeability-surface (PS) area product [42]. As glutamine contributes to the production of adenosine triphosphate (ATP), the biosynthesis of macromolecules and the modulation of redox homeostasis, it is important for the survival of tumor cells [43]. Tumor cells can not only obtain glutamine from plasma but also can synthesize glutamine by themselves intracellularly. GS is the only enzyme known which can convert ammonia and glutamate to glutamine in the mammalian brain tissue [39]. Since the up-regulation of GS is widely interpreted as a reflection of active glutamine metabolism and the up-regulation has been reported in many tumors [42, 44–46], the expression of GS is another factor contributing to the ^13^N-ammonia trapping, that is, metabolic trapping [47–49]. In our study, meningiomas exhibited extremely high accumulation of ^13^N-ammonia against the surrounding tissue because of the absence of BBB, increased regional CBF coupled with increased PS (due to neovascularization), indicating that ^13^N-ammonia is an ideal tracer to identify meningiomas. Immunohistochemical staining of brain tumor biopsies indicated that the GS activity of meningiomas was strongly positive and the expression was not limited to any particular histopathological variants. Amongst the tumors the highest levels were found in the astrocytoma and oligodendroglioma and GS level was higher in meningiomas than pituitary adenomas [39, 46]. Similarly, the normal pituitary tissue clearly showed obviously high uptake of ^13^N-ammonia. Previous study also demonstrated that GS activity was present in the anterior pituitary gland [50]. Since ^13^N-ammonia PET/CT imaging is valuable in detecting pituitary tissue, surgeons can carefully find the pituitary tissue guided by PET so that maximize the protection of the pituitary tissue. This is another reason for the use of ^13^N-ammonia in the sellar region tumors [51]. For NFPMAs, relatively poor neovascularization compared with SSMs and normal pituitary tissue was confirmed on contrast CT and MRI imaging, providing a limited amount of radiotracer for trapping. From the visual results, we found there were uptake overlap between NFPMAs and SSMs for each tracer. The combination of ^18^F-FDG and ^13^N-Ammonia PET/CT could derive a more favorable results for increasing the accuracy to the maximum.

Our study yielded significant results and proposed a new viewpoint for the prediction of SSMs. However, it was inevitable that there were several limitations. Firstly, we collected a small sample of SSMs, and the research results need further confirmation by prospective studies with larger sample capacity. In addition, ^13^N-ammonia has a very short half-life time (9.965 min), so a cyclotron onsite is required for clinical application. Lastly, we failed to detect the GS expression in meningioma specimens limited to the access to resources.

## Conclusion

SSMs and NFPMAs have different metabolic characteristics on ^18^F-FDG and ^13^N-ammonia PET images. The combination of these two tracers can effectively distinguish SSMs from NFPMAs.

## Data Availability

The dataset supporting the conclusions of this article is included within the article. Data and materials during the current study are available from the corresponding author upon reasonable request.

## Abbreviations

SSMs: Suprasellar meningiomas
NFPMAs: Non-functioning pituitary macroadenomas
PET: Positron emission tomography
CT: Computed tomography
T/G: Tumor to normal gray matter
MRI: Magnetic resonance imaging
Gd-DTPA: Gadolinium-Diethylenetriaminepentaacetic acid
GS: Glutamine synthetase
SUV_max_: Maximum standard uptake value
ROI: Region of interest
PWI: Perfusion-weighted imaging
BBB: Blood brain barrier
CBF: Cerebral blood flow
PS: Permeability-surface
GS: Glutamine synthetase
ATP: Adenosine triphosphate
